# Does a prior hysterectomy complicate transvaginal/transumbilical hybrid NOTES cholecystectomy?—a comparative analysis of prospectively collected data

**DOI:** 10.1007/s00423-021-02401-8

**Published:** 2021-12-29

**Authors:** Dirk R. Bulian, Axel Sauerwald, Panagiotis Thomaidis, Claudia S. Seefeldt, Dana C. Richards, Sissy-A. Schulz, Niklas J. Weltermann, Markus M. Heiss, Claus F. Eisenberger

**Affiliations:** 1 Department of Abdominal, Tumor, Transplant and Vascular Surgery, Cologne-Merheim Medical Center, Witten/Herdecke University, Ostmerheimer Strasse 200, D-51109 Cologne, Germany; 2 Department for Obstetrics and Gynecology, Holweide Hospital, Neufelder Strasse 32, D-51067 Cologne, Germany; 3 Department of Gynecology and Obstetrics, St. Marien- Hospital, Hospitalstraße 44, D-52353 Dueren, Germany

**Keywords:** Transvaginal NOTES, Cholecystectomy, Outcome, Hysterectomy, Complication rate, Colpotomy

## Abstract

**Purpose:**

Hysterectomy alters the anatomy of the posterior vaginal vault used as access for transvaginal/transumbilical hybrid NOTES cholecystectomy (NC), creating potential consequences for the feasibility and complication rate of the procedure. Therefore, the aim of our retrospective analysis of prospectively collected data was to analyze the postoperative course after NC in previously hysterectomized (PH) patients compared with patients who had not undergone hysterectomy (NH).

**Methods:**

A total of 126 NH patients and 50 PH patients aged over 42 who had an NC from 12/2008 to 04/2021 were compared regarding age, body mass index (BMI), ASA classification, number of percutaneous trocars, need for intraoperative urinary bladder catheterization, length of procedure, conversion rate, and intraoperative and postoperative complication rate according to the Clavien/Dindo classification, Comprehensive Complication Index (CCI), mortality, and hospital length of stay.

**Results:**

PH patients were older than NH patients (63.0 vs 51.5 years; *P* < 0.001) but did not differ significantly in ASA classification (*P* = 0.595) and BMI (26.8 vs 27.9 kg/m^2^; *P* = 0.480). They required more percutaneous trocars (*P* = 0.047) and longer procedure time (66.0 vs. 58.5 min; *P* = 0.039). Out of all 287 scheduled NC only one had to be “converted” to traditional laparoscopic cholecystectomy. Intraoperative and postoperative complication rates, Clavien/Dindo classification, CCI, need for intraoperative urinary bladder catheterization, and length of stay did not differ significantly.

**Conclusion:**

Our results indicate an increased degree of difficulty of NC in PH patients, although there is no major impact on intraoperative and postoperative complication rates. Urinary bladder perforation is a specific access-related complication in PH patients.

**Supplementary Information:**

The online version contains supplementary material available at 10.1007/s00423-021-02401-8.

## Introduction

Traditional laparoscopic cholecystectomy (LC) is the gold standard in the treatment of gallstone disease in many countries. Transvaginal hybrid NOTES cholecystectomy (NC), developed as an alternative procedure, has advantages over LC by reducing postoperative pain and postoperative analgesic requirements while accelerating postoperative convalescence and improving the aesthetic surgical outcome without increasing the intraoperative or postoperative complication rate [1]. Access to the abdominal cavity in transvaginal hybrid NOTES cholecystectomy is through the posterior vaginal vault into the pouch of Douglas, which is relatively easy to perform [2]. Access-related complications are rare, but rectal or urinary bladder injuries, for example, may occur [3]. However, these complications are only expected after hysterectomy (PH), as the urinary bladder is otherwise separated from the posterior vaginal vault by the portio, cervix, and the uterus. In addition, after a previously performed hysterectomy, adhesions may make transvaginal access difficult or even impossible. These circumstances could signify a higher degree of difficulty of the procedure and a potentially higher risk of access in PH patients. However, to date, only one small case series describing transvaginal NOTES access after hysterectomy for gynecologic surgery predominantly for ovarian cysts has been published [4]. This issue has not yet been investigated for NOTES cholecystectomies. Therefore, we comparatively analyze the data of our NC patients, stratified by their hysterectomy experiences, in order to detect differences in the intraoperative and postoperative course.

## Material and methods

### Patients

**Fig. 1:**
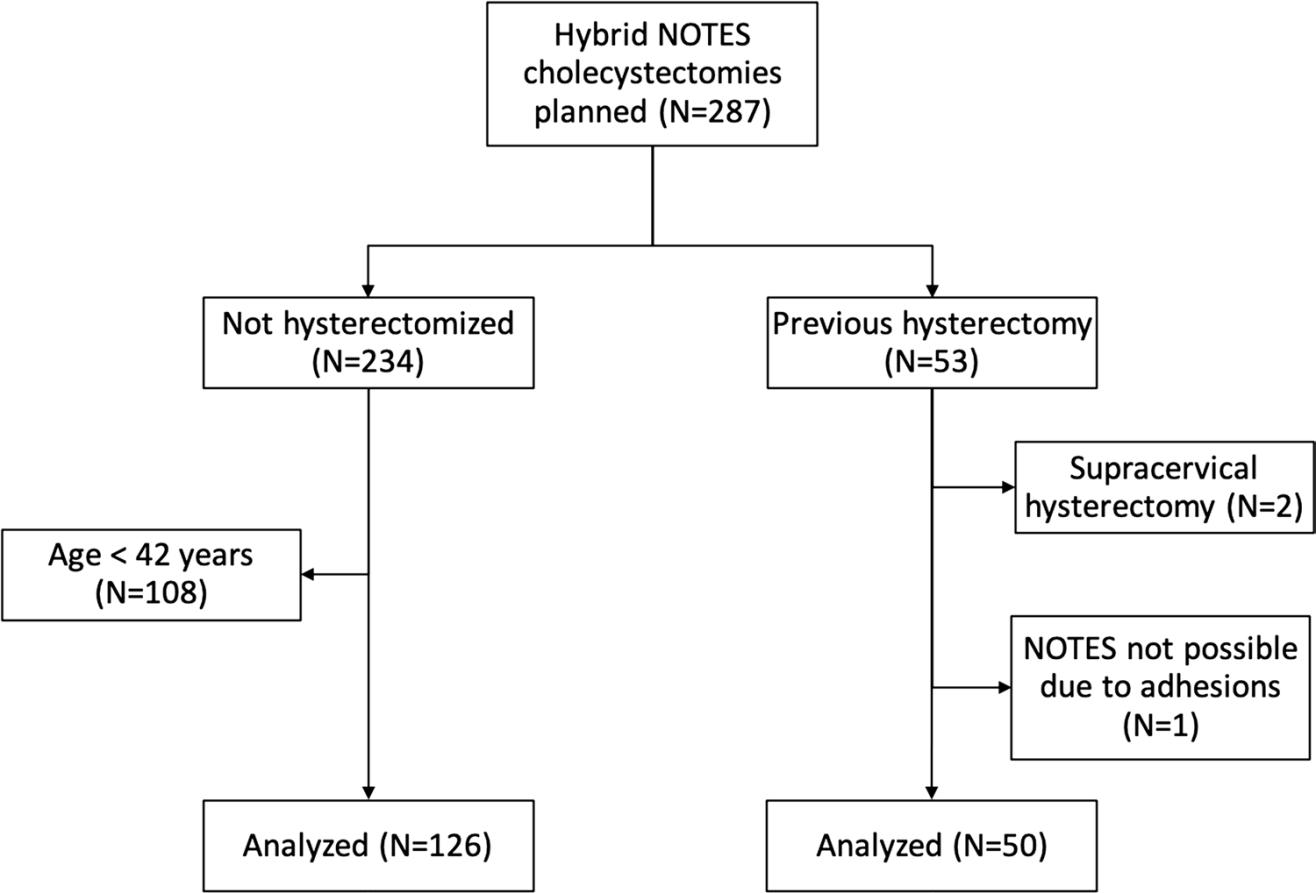
Trial flow diagram

In the period between December 2008 and April 2021, 287 transvaginal/transumbilical cholecystectomies were scheduled in our hospital, Kliniken der Stadt Köln (Fig. 1). Of these 287 patients, 53 had a post-hysterectomy condition, in which we made no distinction between abdominal and vaginal hysterectomy. However, two of these patients had undergone supracervical hysterectomy, which meant that the portio and cervix were still in situ in these patients. Since in these cases the anatomy was unchanged with respect to the posterior vaginal vault and the separation of the posterior vaginal vault to the urinary bladder during the transvaginal approach, the two patients were excluded from the analysis. In one hysterectomized patient, extensive adhesions in the lower right abdomen were found in the initial transumbilical laparoscopy which were most likely attributable to the condition after appendectomy in a case of perforated appendicitis with extensive peritonitis. The adhesions prevented a transvaginal approach to the upper abdomen, so a traditional laparoscopic cholecystectomy was performed in this patient. After excluding this patient as well, 50 PH patients remained for analysis. Looking at the control group of 234 non-hysterectomized patients (NH), a clear skew in the age distribution of the two comparison groups becomes apparent. This is plausible, since a hysterectomy is mainly performed at an advanced age. The age distribution and the difference in this respect are shown in Fig. 2a and b (Electronic Supplementary Material). Since the youngest PH patient was 42 years old, all patients younger than 42 years were excluded for the control group. With this parallelization, we tried to reduce the heterogeneity of both groups [5]. After parallelization, 126 patients remained in the control group.

### Surgical technique

The technique of transvaginal/transumbilical hybrid NOTES cholecystectomy has been performed unchanged throughout the study period and has been described previously [6]. In patients with unchanged anatomy, the transvaginal approach was performed, after creating a capnoperitoneum and transumbilical diagnostic 5 mm laparoscopy, and exposing the portio using vaginal specula (according to KRISTELLER, 110 × 36 mm, 220 mm, Aesculap AG, Tuttlingen, Germany). With an uterus probe (according to SIMS, 330 mm, 4 mm, Aesculap AG, Tuttlingen, Germany) inserted through the portio, the uterus is anteflectively exposed so that the posterior vaginal vault is clearly visible vaginally as well as abdominally. Under diaphanoscopy and laparoscopic visual control, the posterior vaginal vault is perforated with the extra-long 5-mm trocar mandrel (17 cm, Karl Storz GmbH & Co KG, Tuttlingen, Germany) and the curved 5-mm grasping forceps (according to CUSCHIERI O-CON, 43 cm long, Karl Storz GmbH & Co KG, Tuttlingen, Germany) is inserted over it intraabdominally. Directly adjacent, an 11-mm trocar without a connector for insufflation (15 cm, Karl Storz GmbH & Co KG, Tuttlingen, Germany) is also inserted transvaginally to intraperitoneal under visualization.

In the case of a condition after hysterectomy, the vaginal stump is exposed and pressed slightly intraabdominally with the vaginal specula after the creation of the capnoperitoneum and transumbilical diagnostic 5-mm laparoscopy. Then, directly dorsal to the vaginal stump scar, the possible access is palpated from vaginally with closed forceps if the access cannot already be viewed diaphanoscopically. If the route appears clear, the posterior vaginal wall is perforated with the extra-long 5-mm trocar mandrel and the curved 5-mm grasping forceps and 11-mm trocar are inserted as described above. Closure of the transvaginal access at the end of the operation is performed with a continuous, single-row, full-thickness suture with an absorbable braided 0 suture (poly(glycolide-co-l-lactide 90/10); Novosyn®, B. Braun, Melsungen, Germany) and does not differ between the two patient groups, nor does the postoperative treatment.

### Gynecological involvement

For the first 16 transvaginal/transumbilical hybrid NOTES cholecystectomies, the transvaginal approach was performed by a gynecologist, after which the access was also performed by the surgeon.

Routinely, the patients had pre (up to four weeks) and postoperative (about 14 days) gynecological examination.

### Outcome parameter

Patient-side parameters were age, height, weight, the American Society of Anesthesiologists (ASA) classification, and acute cholecystitis. For the outcome parameters, we analyzed the number of required percutaneous trocars, the conversion rate, necessary urinary catheterization, length of procedure, intraoperative and postoperative complications, postoperative length of stay, and mortality. Postoperative complications were classified and compared according to Clavien/Dindo [7]. In addition, we calculated the comprehensive complication index (CCI) [8]. The CCI reflects the overall postoperative morbidity and its severity and ranges from 0 (no complication) to 100 (death). To generate the CCI, we applied the calculator available online (http://www.assessurgery.com).

Most of the parameters were prospectively entered into a registry from the beginning on, and the first 29 patients were missing an ASA classification.

The patient-side parameters were used to test the comparability of the two patient groups. The length of procedure, the conversion rate, the need for urinary bladder catheterization, and the number of required percutaneous trocars were used to test for a different degree of difficulty of the procedure, and the remaining parameters were used to test for a different complication severity of the procedure.

### Statistics

The data were prepared in Microsoft Excel, and SPSS Statistics 28 (IBM Corp., Armonk, NY, USA) was used for the statistical analysis and data processing of all variables. Data of continuous variables are expressed as minimum, maximum, and median. Binary and categorical variables are reported as counts and percentages. The Mann–Whitney *U* test was used for continuous parameters, the Chi-square test for categorical parameters, and the Chi-square test for trend for ordinally scaled variables. A value of *p* < 0.05 was considered statistically significant.

## Results

Before parallelization, the median age of the NH group was 43.5 years, while the youngest PH patient was 42 years old. After excluding NH patients younger than 42, the median age in the control group was 51.5 years, which was still significantly lower than in the PH group, since the age distribution in the parallelized comparison group is significantly shifted to the left, as shown in Fig. 2c (Electronic Supplementary Material). However, the other patient-side parameters of height, weight, BMI, and ASA classification were not significantly different (Table 1), so outcome parameters were analyzed between these two groups. The proportion of acute cholecystitis was almost identical in both groups. There were also hardly any patients with ongoing anticoagulation in either group, so no bias regarding different levels of difficulty between the two groups was expected in this regard. The number of percutaneous trocars used and the length of procedure were found to be significantly different, whereas the need for intraoperative urinary bladder drainage, the intraoperative as well as postoperative complication rate, the Clavien-Dindo classification of postoperative complications, and the Comprehensive Complication Index did not differ significantly (Table 2).

**Table 1:** Baseline characteristics of all patients. Values are reported as median (min – max) and counts (percentage)

Variable	NH (*n* = 126*)	PH (*n* = 50*)	Total (*n* = 176*)	*P* value
Age [years]	51.5 (42–86)	63.0 (42–85)	55.0 (42–86)	< 0.001
Height [cm]	165 (150–181)	165 (150–175)	165 (150–181)	0.624
Weight [kg]	75 (53–171)	70 (50–114)	75 (50–171)	0.350
BMI [kg/m^2^]	27.9 (18.0–52.2)	26.8 (18.7–43.3)	27.3 (18.0–52.2)	0.480
ASA*				0.595
1	10 (9.5)	2 (4.8)	12 (8.2)	
2	76 (72.4)	33 (78.6)	109 (74.1)	
3	19 (18.1)	7 (16.7)	26 (17.7)	
Acute cholecystitis—–yes	12 (9.5)	4 (8.0)	16 (9.1)	1.000

*NH* not hysterectomized, *PH* previous hysterectomy, *BMI* body mass index, *ASA* Classification of the American Society of Anesthesiologists (*available for 105 NH and 42 PH patients)

**Table 2:** Patient outcome

Variable	NH (*n* = 126)	PH (*n* = 50)	Total (*n* = 176)	*P* value
No. of percutaneous trocars	1 (1–4)	1 (1–3)	1 (1–4)	0.047
1	93 (73.8)	30 (60.0)	123 (69.9)	
2	29 (23.0)	15 (30.0)	44 (25.0)	
3	3 (2.4)	5 (10.0)	8 (4.5)	
4	1 (0.8)	0	1 (0.6)	
Conversion—yes	0	0	0	
Urinary catheter used*—yes	47 (48.0)	24 (60.0)	71 (51.4)	0.260
Length of procedure [minutes]	58.5 (29–135)	66.0 (25–150)	60 (25–150)	0.039
Intraoperative complications—yes	1 (0.8)	1 (2.0)	2 (1.1)	0.489
Postoperative complications—yes	11 (8.7)	3 (6.0)	14 (8.0)	0.760
Clavien-Dindo classification of postoperative complications				0.990
No complication	115 (91.3)	47 (94.0)	162 (92.0)	
Grade I	5 (4.0)	0	5 (2.8)	
Grades II	3 (2.4)	1 (2.0)	4 (2.3)	
Grades III	3 (2.4)	2 (4.0)	5 (2.8)	
Comprehensive Complication Index	0 (0–33.7)	0 (0–33.7)	0 (0–33.7)	0.595
Postoperative hospital stay [days]	2 (1–14)	2 (1–12)	2 (1–14)	0.624
Mortality—yes	0	0	0	

Values are reported as median (min – max) and counts (percentage). *NH* not hysterectomized, *PH* previous hysterectomy

^*^Urinary catheter used was available for 138 patients

The conversion rate and mortality were zero in both groups.

The only intraoperative complication in the PH group was urinary bladder perforation due to an 11 mm trocar inserted too far ventrally, which was already detected intraoperatively. This case was the first time NC was performed in a PH patient in whom transvaginal access was established by a gynecologist. The intraoperative suspicion of urinary bladder perforation was confirmed laparoscopically by leakage of methylene blue after filling the urinary bladder via an inserted catheter. The exit lesion of the urinary bladder was closed laparoscopically before completion of the procedure after placing two additional percutaneous 3-mm and 5-mm trocars, respectively, with 2–0 Novosyn® single button sutures (poly(glycolide-co-l-lactide 90/10); B. Braun, Melsungen, Germany), and the entry lesion of the urinary bladder was closed transvaginally with the same suture material. Repeated blue filling of the urinary bladder then confirmed the tightness of both sutures. In addition, an intraoperative cystoscopy was performed to exclude an injury of the ostia ureterum or the ostium urethrae internum. The postoperative course was unremarkable with the catheter in place; the catheter was removed after cystography on postoperative day 12, and the patient was discharged the following day.

This complication did not recur in the subsequent 49 PH patients.

The only intraoperative complication in the NH group was venous hemorrhage in the gallbladder bed, which was controlled by argon beamer with the aid of two additional transcutaneous 5-mm auxiliary trocars. Again, the postoperative hospital stay was prolonged at six days. No other access-related complications, such as rectal or colonic injury, occurred in our patients.

Details of patients with postoperative complications are shown in Table 3.

**Table 3:** Postoperative complications

Patient no	NH/PH	Age	BMI	ASA	Postoperative complication details	Clavien-Dindo	CCI	LoP	LoS
39	NH	61	30.5	2	Postoperative biliary pancreatitis	2	20.9	42	14
42	NH	71	23.1	2	Newly occurred tachyarrhythmia in cases of atrial fibrillation	2	20.9	59	6
58	NH	74	22.3	2	Infected hematoma in the former gallbladder bed	2	20.9	77	3
70	NH	52	24.9	2	Perioperative stone passage with consecutive cystic stump insufficiency	3	26.2	74	7
90	NH	84	25.4	2	Hematoma in the former gallbladder bed	3	33.7	101	8
92	NH	75	29.5	3	Postoperative ileus	1	8.7	68	8
95	NH	50	25.5	2	Subhepatic hematoma, anemia	1	8.7	87	3
129	NH	61	28.1	2	Febrile nasopharyngeal infection	1	8.7	73	3
130	PH	73	28.7	2	PONV, unclear CRP increase	2	22.6	76	5
135	NH	51	46.3	3	Vertigo, cold with sore throat and feeling of pressure on the chest	1	15.0	85	4
144	NH	55	27.3	2	Cystic stump insufficiency	3	33.7	100	7
158	NH	71	32.4	3	Vaginal smear bleeding under therapeutic anticoagulation	1	8.7	64	2
168	PH	61	38.1	3	Vaginal wound bleeding	3	33.7	60	2
176	PH	51	24.1	2	Postoperative choledocholithiasis	3	26.2	51	4

*NH* not hysterectomized, *PH* previous hysterectomy, *BMI* body mass index, *ASA* Classification of the American Society of Anesthesiologists, *CCI* Comprehensive Complication Index, *LoP* length of procedure [minutes], *LoS* postoperative length of stay [days], *PONV* postoperative nausea and vomiting, *CRP* C-reactive protein

Of the patients with more than one percutaneous trocar, the insertion of the further trocars was significantly more frequently performed before the transvaginal approach in the PH group (6/20 patients, 30%) than in the NH group (2/33 patients, 6.1%; *P* = 0.042). More than one percutaneous trocar was necessary in the two NH patients before the transvaginal approach for anteflexion of the uterus to expose the pouch of Douglas. In most of the PH patients with pre-transvaginal approach insertion of further trocars, it was due to adhesions after the hysterectomy 5/6 patients. Most of the other patients of both groups, the need of further trocars after the transvaginal approach was due to pericholecystic adhesions or for dissection of the gallbladder.

## Discussion

The aim of our study was to analyze the influence of having had a hysterectomy on the difficulty level and complication rate of NC. According to our literature search, this is the first comparison of intraoperative and postoperative parameters after NOTES procedures in general surgery in patients with and without a previous hysterectomy. Because a prospective randomized study is not possible for this question, so we have to resort to a cohort comparison.

Surgery via natural orifices was developed to avoid the access trauma and complications of accessing the abdominal cavity via the abdominal wall, such as wound infections, wound dehiscence up to burst abdomen, and scar hernias. In this regard, the transvaginal approach via the posterior vaginal vault is an obvious choice, as it was developed in gynecology a long time ago [9]. However, the approach was unknown in abdominal surgery for a long time until Delvaux et al. published six cases of removal of the gallbladder with large stones (diameters of 4 to 6 cm) after laparoscopic cholecystectomy via the posterior vaginal vault in 1993 [10]. One of the six patients had undergone hysterectomy 7 years earlier, so that in this case access for gallbladder removal had to be via the vaginal stump with altered anatomy. According to our literature search, this is the first described case in which a gallbladder was retrieved transvaginally in a patient with a previous hysterectomy. One year later, Zornig’s group described transvaginal specimen removal in two cases of laparoscopic colon surgery and in 1995 the case of transvaginal splenic removal [11, 12]. In 1999, Tsin et al. described a hybrid NOTES cholecystectomy in which, after performing a vaginal hysterectomy, a 12-mm trocar was inserted via the vaginal stump, sealed by means of a tabac pouch suture, and a laparoscope was inserted to the upper abdomen. Additional 5-mm trocars were used to remove the gallbladder and retrieve it via the vaginal approach [13]. However, access via the posterior vaginal vault is much easier when the uterus is still present and removal of fibroids up to 13 cm in size via culdotomy while maintaining their integrity has been described [14]. In 2010, Zorron et al. considered a previous hysterectomy as a contraindication for transvaginal access in their IMTN study [15], even though Zornig et al. already operated on a patient with a previous hysterectomy in their series of the first 20 NC 2007 [16].

A complication specific to transvaginal access after hysterectomy for the aforementioned anatomic reasons is urinary bladder injury, which cannot occur in NH patients. In the first large registry analysis of NOTES procedures, 4 urinary bladder injuries (0.7%) were found in 547 transvaginal procedures, although it was not recorded how many patients had a previous hysterectomy [3]. Three of these had only been treated with an inserted bladder catheter. An updated analysis of the German NOTES Registry showed eleven bladder injuries in 2928 transvaginal accesses (0.4%), again with no indication of the proportion of PH patients [17]. In a review of the Swiss Association for Laparo- and Thoracoscopic Surgeons database, one urinary bladder injury was found in 454 transvaginal procedures (0.2%), necessitating an overstitch [18]. Thus, no real complication rate of urinary bladder injury in PH patients can be reported. In our analysis, one case with such a complication was found in 286 NC performed, i.e., in 0.3%. This is in alignment with the results from the literature. However, if we take the number of PH patients as the denominator, the complication rate in our study is 2%. It is interesting that this specific complication occurred with the first NC of a PH patient and not again afterwards. Also, the complication rate decreased over time in the German NOTES Registry from 0.7 to 0.4%, which could also be attributed to a possible learning curve in this regard. In particular, also because the rate of PH patients may have increased over time, since, as recommended by Zorron et al., a previous hysterectomy was initially seen as a contraindication for transvaginal access rather than in the further course. Thus, in the case of a stable correlation between hysterectomy performed and intraoperative urinary bladder injury, an increasing complication rate would be expected, but this is not present.

We did not distinguish between abdominal and vaginal hysterectomy because the relatively small group size did not allow for further subgroup analysis.

Another access-specific but hysterectomy-independent intraoperative complication documented in the 2015 German NOTES Registry was bowel injury in 0.2% (five of 2625 cases). This complication did not occur in our hospital or in the analysis of the Swiss database. Overall, our intraoperative complication rate was 2% in the PH group, 0.8% in the NH group, and 1.1% in the entire collective. The intraoperative complication rate was 1.6% in the German registry and 0.7% in the Swiss database, both without stratification by hysterectomy.

In our patient collective, 14 postoperative complications (8.0%) were found, two of which were access-specific (1.1%). Both cases involved vaginal rebleeding, one of which was treated conservatively and one surgically. One occurred in a PH patient and the other in a NH patient. This complication was detected in 0.2% of patients in the analysis of the Swiss database and in 0.3% in the German NOTES Registry. Of the eight vaginal rebleeds in the German NOTES Registry, 75% could be treated conservatively.

Our analysis evaluates for the first time intraoperative and postoperative parameters after NC in relation to a previous hysterectomy and reveals a significantly longer operative time and a significantly more frequent use of additional transcutaneous auxiliary trocars after hysterectomy. This can most likely be interpreted as a reflection of the increased difficulty of transvaginal access in this patient group. The complication rate was not increased; however, evaluation of the complication rate is limited because of the small number of cases. We identified one complication occurring only after hysterectomy; it was a urinary bladder injury, which healed with appropriate treatment and did not cause permanent damage.

In our experience, we recommend that transvaginal access in PH patients should be only performed under direct laparoscopic view of the vaginal stump and, if necessary, after appropriate adhesiolysis to avoid urine bladder injuries. The access should be made as far dorsal as possible, but respecting the rectum. It should also be noted that the resistance of the vaginal stump is often significantly greater than the resistance of the posterior vaginal vault in NH patients. In the case of sudden loss of resistance, care must then be taken to avoid inadvertent injury to the rectum or small intestine with the trocar mandrel or trocar.

The major weakness of our study is the insufficient number of cases for a robust significance analysis of intraoperative and postoperative complications. Here, a multicenter evaluation is necessary but has not been possible so far because of the lack of survey data on hysterectomy status in the national and international databases. The second weakness is the different age distribution in the two comparison groups due to the performance of hysterectomy at an advanced age. However, there is no evidence that patient age is an independent influencing parameter on operative time and need for additional percutaneous trocars. Here, a different study design such as matched-pair analysis or multivariate analysis would help, but both would require much larger comparison groups.

## Conclusion

We compared the length of procedure, number of percutaneous trocars, intraoperative and postoperative complication rates, and hospital length of stay after NC between patients after hysterectomy with those without prior hysterectomy. We interpret the significantly prolonged length of procedure and significantly increased number of percutaneous trocars as a sign of the higher complexity of the procedure in PH patients, but the non-increased intraoperative and postoperative complication rate and especially the non-significantly different postoperative hospital length of stay speak to the feasibility of the procedure in this patient group. However, urinary bladder injury should be mentioned as a specific complication in post-hysterectomy patients. Therefore, the procedure in PH patients should only be performed in centers with sufficient experience.

## Supplementary Information

Below is the link to the electronic supplementary material.

Supplemental Fig. 1 Age Distribution of all nonhysterectomized patients (PNG 24.8 KB)

High resolution image (EPS 28.4 KB)

Supplemental Fig. 2 Age Distribution of the hysterectomized patients (PNG 23.7 MB)

High resolution image (EPS 28.5 KB)

Supplemental Fig. 3 Age Distribution of the nonhysterectomized patients over 41 years of age (PNG 21.2 MB)

High resolution image (EPS 33.7 KB)

## Data Availability

The authors confirm that the data supporting the findings of this study are available within the article.
