# Supplementary figures and images for: Does a prior hysterectomy complicate transvaginal/transumbilical hybrid NOTES cholecystectomy?—a comparative analysis of prospectively collected data
Source: Langenbecks Arch Surg. 2021 Dec 29;407(2):655–62. doi: 10.1007/s00423-021-02401-8 (PMC8933306; doi:10.1007/s00423-021-02401-8)

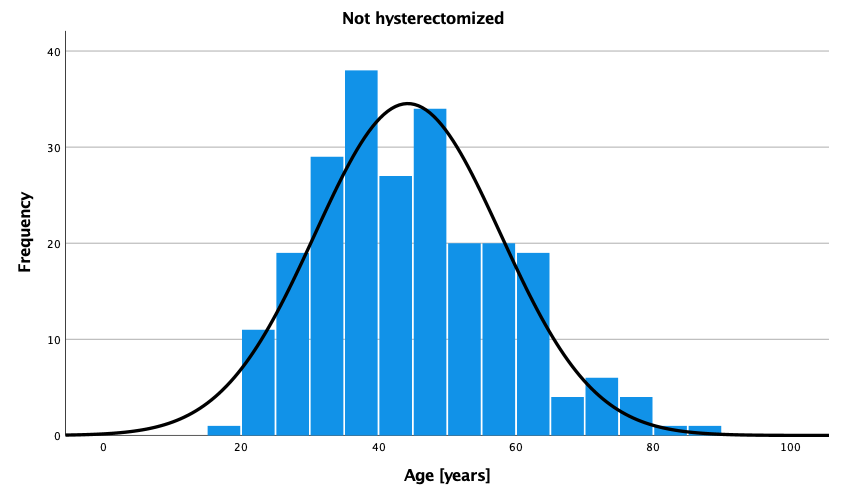

Supplement: Supplementary file 1 — Age Distribution of all nonhysterectomized patients (PNG 24.8 KB) [file 423_2021_2401_Fig2_ESM.png]

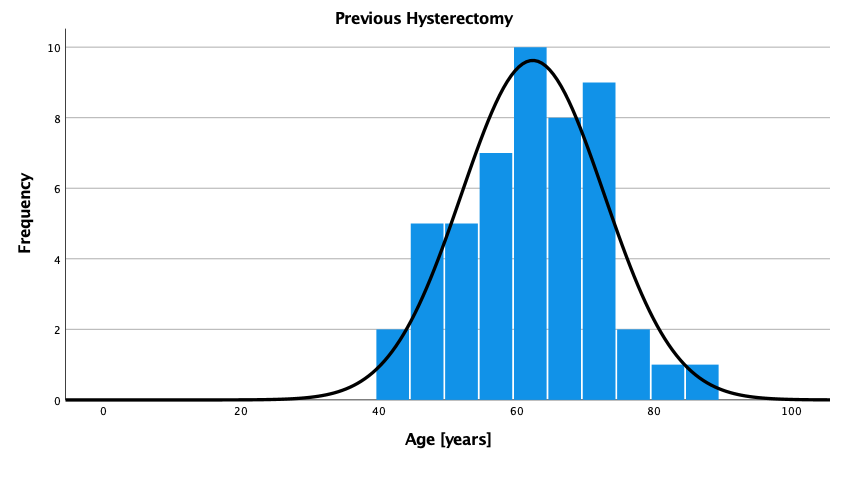

Supplement: Supplementary file 3 — Age Distribution of the hysterectomized patients (PNG 23.7 MB) [file 423_2021_2401_Fig3_ESM.png]

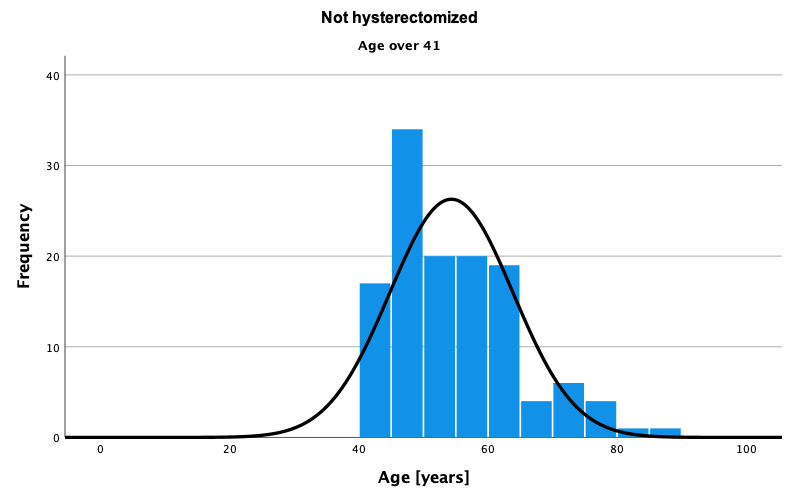

Supplement: Supplementary file 5 — Age Distribution of the nonhysterectomized patients over 41 years of age (PNG 21.2 MB) [file 423_2021_2401_Fig4_ESM.png]
